# A Chinese survey of clinical practice on the management of thyroid eye disease

**DOI:** 10.1530/ETJ-23-0269

**Published:** 2024-05-23

**Authors:** Jingyue Chen, Chenyan Li, Weiping Teng, Zhongyan Shan, Jun Jin, Yining Wei, Jing Sun, Yushu Li, Huifang Zhou

**Affiliations:** 1 Department of Endocrinology and Metabolism, Institute of Endocrinology, NHC Key Laboratory of Diagnosis and Treatment of Thyroid Diseases, The First Affiliated Hospital of China Medical University, Shenyang, China; 2 Department of Ophthalmology, Shanghai Ninth People's Hospital, Shanghai Jiao Tong University School of Medicine, Shanghai, China

**Keywords:** clinical management, clinical survey, endocrinology, ophthalmology, questionnaire, thyroid eye disease

## Abstract

**Objective:**

The management of thyroid eye disease (TED) has undergone significant changes for decades. The study sought to investigate current clinical practice on the management of TED in China.

**Methods:**

An online questionnaire survey was conducted from April to May 2023. The questionnaire involved diagnostic criteria for TED, multidisciplinary treatment (MDT) collaboration, and treatment preference for mild, moderate, and severe TED.

**Results:**

A total of 289 questionnaires were collected, with 165 from endocrinologists and 124 from ophthalmologists. Only 36.7% of participants claimed there was an MDT clinical pattern for TED in their institutions. The coverage of biological agents was around 10% or lower. These were distinctly lower than in Western countries. About 62.6% of participants believed the incidence of TED has increased in recent years. Imaging techniques were used widely to assist in the diagnosis of TED. However, there was still controversy regarding the definition of proptosis in the Chinese population. Most doctors managed risk factors and provided orbital supportive treatments of artificial tears and glasses. For mild active TED, endocrinologists (39.4%) were inclined to recommend therapy for hyperthyroidism alone, while ophthalmologists (43.6%) preferred orbital corticosteroid injections. Currently, the most widely used treatment for moderate to severe active TED was high-dose intravenous corticosteroid (94.8%), while orbital radiotherapy combined with immunosuppressive agents was the most recognized second-line therapy (43.6%).

**Conclusion:**

The study documented the consistency and differences between current clinical practices in the management of TED in China and the recently updated guidelines. There was a remarkable difference between ophthalmology and endocrinology departments, warranting management optimization.

## Introduction

Thyroid eye disease (TED), also known as thyroid-associated ophthalmopathy or Graves’ ophthalmopathy, is an infiltrative disorder affecting the retro-orbital and peri-orbital tissues caused by autoimmune system dysfunction. It affects ~30% of patients with Graves’ disease, although it may pass unnoticed in a milder form (1). The most obvious and visually striking symptom of TED is proptosis, although some patients may not exhibit this symptom but experience severe visual impairment. Other common symptoms include photophobia, tearing, conjunctival and eyelid congestion and swelling, restricted eye movement, and restrictive strabismus (2, 3). TED not only poses a physical burden but also leads to psychological problems. Patients with TED face obstacles, such as unemployment, reduced income, and loss of quality of life (4, 5), leading to depression, irritability, and even suicidal behavior (6, 7). Therefore, TED deserves attention and requires timely care and treatment.

In 2008, the European Group on Graves’ Orbitopathy (EUGOGO) released a consensus statement on the treatment of Graves’ Orbitopathy (GO) (8). In 2016, the European Thyroid Association (ETA) collaborated with EUGOGO to publish the management guidelines for GO (9). The guidelines were updated in 2021 (10). In 2022, the American Thyroid Association (ATA) and the ETA jointly published a consensus statement for TED (11). These guidelines and consensus statements provide reliable and referenceable documents for healthcare professionals and patients worldwide. Building upon this, Chinese scholars have published interpretations of the latest version of the EUGOGO guidelines and the ‘Chinese guideline on the diagnosis and treatment of thyroid-associated ophthalmopathy’ (12, 13).

Currently, high-dose intravenous corticosteroid therapy is recommended as a first-line treatment for moderate-to-severe active TED in China, inevitably resulting in a series of side effects such as osteoporosis, infections, and elevated blood sugar levels (14, 15). The immunosuppressive agents (e.g. mycophenolate) in combination with intravenous corticosteroids are more effective than corticosteroids alone (16). In recent years, new drugs have been developed and gradually introduced into clinical practice. Teprotumumab (insulin-like growth factor-1 receptor (IGF-1R) antibody) has been proven to significantly reduce clinical activity score (CAS) and proptosis and improve diplopia (17, 18, 19). In patients with moderate-to-severe active TED, both tocilizumab (interleukin-6 antibody) and rituximab (CD20 antibody) have also shown efficacy in alleviating disease manifestations and reducing CAS scores (20, 21, 22, 23, 24). These represent significant advancements in the field of autoimmune thyroid disorders over the past 20 years (25).

However, the extent to which Chinese clinical practices align with these management guidelines for TED remains unknown. Endocrinology and ophthalmology departments play a crucial role in TED management and are often the initial place where patients turn to for assistance. Referring to the guidelines and some international TED management surveys, we formulated a questionnaire about the management of TED, including nearly 40 questions. The survey primarily targeted endocrinologists and ophthalmologists to identify practical differences and difficulties in the diagnosis and treatment of TED.

## Materials and methods

### Survey topic determination and questionnaire design

Prior to formulating the questionnaire, we conducted an unsystematic search on clinical practices of TED, identifying a total of three studies conducted in the past 5 years. In 2020, two surveys on TED management targeting oculoplastic surgeons were published, one by Pradhan in India (26) and the other by Lee in the United Kingdom (27). In 2022, Brito *et al.* conducted a survey targeting endocrinology specialists for ATA and ETA (28). In addition, a survey on the management of TED was conducted by Chinese scholars in 2015 (29).

We adapted the TED questionnaire content targeting ATA and ETA (28) and added the controversial questions encountered in clinical practice in China, eventually developing our questionnaire. The survey addressed various topics related to TED, including the criteria used for diagnosis, the development of multidisciplinary treatment (MDT), the utilization of orbital imaging, treatment preferences for different levels of TED severity, and concerns regarding current clinical practices. The questionnaire was discussed and decided upon by a small group of members with relevant experience in TED management. All questionnaire were presented and conducted in Chinese. A full description of survey items is provided in Supplementary Appendix Table 1 (see section on supplementary materials given at the end of this article). A brief questionnaire is depicted in Table 1.

**Table 1:** Applicable survey items for management recommendations for TED.

Questions	Response options
GD patients with mild active TED and a CAS of three points present a management challenge. Do you have a preference regarding first-line treatment for hyperthyroidism?	Anti-thyroid drugs, radioiodine, radioiodine plus oral steroid prophylaxis, and thyroidectomy.
For GD patients with mild active TED, what would be your treatment recommendation(s) at this stage for TED? (you may check more than one box if you wish)	Treating hyperthyroidism alone, intravenous corticosteroids, orbital corticosteroids injection, oral corticosteroids, mycophenolate, rituximab, tocilizumab, teprotumumab, rapamycin, cyclosporin, azathioprine, methotrexate, orbital surgery, orbital radiotherapy, and statin therapy.
For patients with moderate-to-severe active TED, which treatment is the first-line treatment?	Intravenous corticosteroids, orbital corticosteroids injection, oral corticosteroids, mycophenolate, rituximab, tocilizumab, teprotumumab, rapamycin, cyclosporin, azathioprine, methotrexate, orbital surgery, and orbital radiotherapy.
Before and after administering intravenous corticosteroids therapy, which indicators would you evaluate in order to prevent adverse reactions in patients? (you may check more than one box if you wish)	Complete blood count, urinalysis, liver function, kidney function, blood glucose, electrolytes, blood coagulation function, indicators related to viral and autoimmune hepatitis, blood pressure, electrocardiogram, chest X-ray or CT scan, and bone density measurement.
What is your most commonly used corticosteroid dosage for moderate-to-severe active TED patients receiving intravenous corticosteroids treatment?	0.5 g/week intravenous methylprednisolone for six consecutive weeks, followed by 0.25 g/week for 6 weeks, 0.5 g/week intravenous methylprednisolone for six consecutive weeks, followed by 0.25 g/week for 6 weeks plus oral mycophenolate, 0.75 g/week intravenous methylprednisolone for six consecutive weeks, followed by 0.5 g/week for 6 weeks, 0.5–1 g/day intravenous methylprednisolone daily or every other day (repeat three times for 1–2 weeks), followed by tapering dose of oral prednisolone
What is your preferred treatment approach for TED patients with DON?	0.75 g/week intravenous methylprednisolone for six consecutive weeks, followed by 0.5 g/week for 6 weeks, 0.5–1 g/day intravenous methylprednisolone daily or every other day (repeat three times for 1–2 weeks), followed by tapering dose of oral prednisolone, urgent eye surgery, and elective eye surgery, 0.5–1 g/day intravenous methylprednisolone daily or every other day (repeat three times for 1–2 weeks), conduct eye surgery if no improvement, tocilizumab as the initial treatment, followed by eye surgery if no improvement.
For type 2 diabetes patients with poor control of blood glucose levels, what treatment(s) would you institute as first line for TED? (you may check more than one if you wish)	Antidiabetic medication plus intravenous corticosteroids, orbital corticosteroids injection, oral corticosteroids, mycophenolate, rituximab, tocilizumab, teprotumumab, rapamycin, cyclosporin, azathioprine, methotrexate, orbital surgery, and orbital radiotherapy.

CAS, clinical activity score; DON, dysthyroid optic neuropathy; GD, Graves’ disease; TED, thyroid eye disease.

### Survey implementation and questionnaire collection

The questionnaire for the diagnosis and treatment of TED was released online during the 12th China Medical University Thyroid Forum and the 14th Shanghai Jiao Tong University Ophthalmology Forum. These two forums are open to endocrinologists and ophthalmologists from all regions in China. All doctors were eligible to participate in the survey voluntarily and anonymously. The forum organizers and authors did not have direct contact with the participants, nor did they provide guidance on participant preferences during the survey period.

Participants completed the questionnaire and uploaded their selections online. Duplicate submissions from the same IP address were automatically blocked. This study was approved by the Ethics Committee of China Medical University. Informed consent was obtained from each participant after a thorough explanation of the purpose and nature of all procedures used in the study.

### Statistical analysis

Due to the setup of the online questionnaire program, respondents’ results were only submitted upon completion of all questions in the survey. Therefore, all data received from the respondents were complete, and there was no missing data.

Statistical analysis with *χ* ^2^ or Fisher’s exact tests for categorical variables was used to assess differences between respondents for the endocrinology and ophthalmology departments. Statistical significance was defined as *P* < 0.05. We used IBM SPSS for statistical analysis.

## Results

### Respondent demographics

The survey invitation was sent to 1566 society members. Of these, a total of 289 respondents, consisting of 165 endocrinologists and 124 ophthalmologists, completed and uploaded the survey (18% response rate). Most of the respondents were from tertiary hospitals (85.5%, 247/289), while 67.8% (196/289) of the respondents were chief physicians or associate chief physicians. The largest number of respondents (33.9%, 98/289) had experience in treating TED for 5–10 years (Table 2).

**Table 2:** Demographics of participants.

	Endocrinology (*n* = 165)	Ophthalmology (*n* = 124)	Total (*n* = 289)
Healthcare institutions			
Tertiary hospitals	138 (83.64%)	109 (87.9%)	247 (85.47%)
Secondary hospitals	22 (13.33%)	10 (8.06%)	32 (11.07%)
Community hospitals	3 (1.81%)	3 (2.42%)	6 (2.08%)
Private hospitals	1 (0.61%)	0 (0%)	1 (0.35%)
Other medical institutions	1 (0.61%)	2 (1.61%)	3 (1.04%)
Professional titles			
Chief physician	63 (38.18%)	20 (16.13%)	83 (28.72%)
Associate chief physician	54 (32.73%)	59 (47.58%)	113 (39.10%)
Attending physician	38 (23.03%)	43 (34.68%)	81 (28.03%)
Resident physician	10 (9.52%)	2 (1.61%)	12 (4.15%)
TED management experience			
Less than 5 years	37 (27.62%)	45 (36.29%)	82 (28.37%)
5–10 years	42 (23.33%)	56 (45.16%)	98 (33.91%)
11–15 years	26 (12.86%)	17 (13.71%)	43 (14.88%)
15–20 years	22 (13.33%)	4 (3.23%)	26 (9.00%)
More than 20 years	38 (22.86%)	2 (1.61%)	40 (13.84%)

### Changes in TED incidence

When queried regarding the annual incidence of TED cases, the respondents’ perspectives were sharply divided. The predominant response indicated encountering 1–5 cases per year (38.8%, 112/289), closely followed by a count exceeding 20 cases (26.64%, 77/289). A majority of doctors (62.6%, 181/289) expressed the belief that the occurrence of new TED cases had witnessed an upward trend, whereas only a small fraction (3.5%, 10/289) of doctors believed in a decrease when compared to previous years.

### Diagnosis and assessment criteria for TED

Given the dissimilarities in facial and orbital structures between Western and Asian populations, it is not advisable to directly employ cutoff values from other countries to evaluate the extent of proptosis among TED patients in China. In most instances, clinical practitioners typically regard a Hertel exophthalmometry measurement 2 mm higher than the previous measurement as indicative of proptosis progression. Nonetheless, this approach relies on the availability of previous exophthalmometry measurement of patients. For newly diagnosed TED patients, we posit the necessity of establishing specific cutoff values for proptosis based on the unique characteristics of the Chinese population.

Significant discordance regarding this issue was observed between ophthalmologists and endocrinologists. The majority of endocrinologists (21.2%, 35/165) identified the cutoff value as 18.3 mm, whereas the greater part of ophthalmologists (33.1%, 41/124) argued for 16 mm. Moreover, 19.0% (55/289) of respondents opted to rely on patients’ self-reported increase in proptosis as a criterion. Furthermore, 11.1% (32/289) of respondents believed that a definitive cutoff value had not yet been established.

Orbital MRI (66.8%, 193/289) and orbital CT (63.7%, 184/289) were currently the prevailing imaging techniques utilized to evaluate the activity and severity of TED. As per the survey, endocrinologists tended to opt for orbital CT (68.5%, 113/165), while ophthalmologists demonstrated a greater preference for MRI (83.9%, 104/124).

In terms of assessing the staging of TED activity, a considerable proportion of doctors, specifically 55.0% (159/289), adopted a combined approach involving the utilization of CAS in conjunction with orbital MRI. Meanwhile, 31.5% (91/289) of doctors relied solely on CAS. It is worth noting that this method carries inherent risks, as CAS provides a close but not entire reflection of disease progression. Regarding the determination of severity grade, the majority of doctors, accounting for 75.1% (217/289), opted for the EUGOGO classification as the standard. More than half the doctors (61.9%, 179/289) claimed they would integrate quality-of-life questionnaire into assessment criteria. The adoption of subjective and objective evaluation is a comprehensive approach.

Regarding the recommended follow-up intervals for moderate-to-severe active TED, whether in the field of endocrinology or ophthalmology, the majority of doctors (52.6%, 152/289) expressed the belief that a monthly follow-up visit was appropriate. Approximately 30.5% (88/289) of doctors recommended a weekly follow-up visit, while fewer participants suggested follow-up visit every 3 months (21.5%, 62/289), at the midpoint of the treatment course (19.0%, 55/289), or at the conclusion of the treatment course (15.2%, 44/289).

### Multidisciplinary treatment of TED

The care of TED requires close collaboration among departments such as endocrinology, ophthalmology, and radiology. Unfortunately, a majority of doctors (63.3%, 183/289), both endocrinologists and ophthalmologists, indicated that their medical institutions lacked an MDT approach to managing TED. Furthermore, 46.0% (57/124) of ophthalmologists consistently and 40.3% (50/124) occasionally referred patients with TED to multidisciplinary clinics. Among endocrinologists, 48.5% (80/165) consistently and 42.4% (70/165) occasionally referred patients with TED to multidisciplinary clinics.

When asked about the timing of referrals to ophthalmology, the responses of endocrinologists varied. About 54.6% (90/165) of endocrinologists immediately referred patients to ophthalmology during the initial visit, while 55.8% (92/165) of endocrinologists referred patients when assessing them as moderate, severe, or extremely severe TED. Only a small percentage of endocrinologists (6.7%, 11/165) opted not to refer patients to ophthalmology.

### Control of risk factors in TED

Several risk factors, such as smoking, lipid levels, vitamin D levels, and selenium levels, hold significance in TED (11). Based on the survey, the prevalence of smoking among TED patients remained high. Approximately 42.2% (122/289) of doctors stated that between 26% and 50% of the newly diagnosed TED patients were smokers, while 19.4% (56/289) of doctors reported that over half of new patients were smokers. Nearly all doctors (94.8%, 274/289) advised TED patients to quit smoking or avoid exposure to smoke.

The majority of doctors recommended lipid level testing in TED patients, but there was a higher proportion of endocrinologists (92.7%, 153/165) compared to ophthalmologists (70.2%, 87/124, *P <* 0.001) who suggested lipid testing. Both specialties recommended the use of lipid-lowering medications for patients with hypercholesterolemia (endocrinologists 96.4% (159/165) vs ophthalmologists 91.1% (113/124)). Endocrinologists (85.5%, 141/165) also had a higher proportion of recommendations for vitamin D supplementation than ophthalmologists (62.1%,77/124, *P <* 0.001) based on the patient’s vitamin D levels.

Among the participants, some (38.4%, 111/289) would advise selenium supplementation for all patients, while others (35.0%,101/289) would only recommend selenium supplementation for patients with mild TED. Additionally, almost all doctors (98.6%, 285/289) would suggest the use of artificial tears or eye ointments, with ophthalmologists having a recommendation rate of 100%. Supportive treatments such as wearing glasses were also commonly recommended, with a rate of 87.9% (254/289).

### Availability of treatment resources

In recent decades, the range of treatment options for TED has expanded significantly. Clinicians can choose the most suitable treatment based on individual characteristics to achieve the optimal benefit-risk ratio. However, due to various factors, not all healthcare institutions have access to all treatment resources.

Currently, the most commonly employed treatments were intravenous corticosteroid therapy (91.4%, 264/289), oral corticosteroids (59.9%, 173/289), and orbital corticosteroid injections (49.8%, 144/289). Orbital surgery (37.7%, 109/289), orbital radiotherapy (24.2%, 70/289), mycophenolate (21.8%, 63/289), rituximab (14.2%, 41/289), methotrexate (13.5%, 39/289), and cyclosporine (13.8%, 40/289) were still available in some medical institutions. However, only a few institutions could offer tocilizumab (9.0%, 26/289), teprotumumab (3.5%, 10/289), rapamycin (1.4%, 4/289), and azathioprine (5.2%, 15/289).

### Management recommendations of TED with different conditions

#### Mild active TED

In the management of patients with mild active TED accompanied by hyperthyroidism, a majority of doctors (endocrinologists 81.8% (135/165) vs ophthalmologists 79.0% (98/124)) would recommend antithyroid drugs to control hyperthyroidism. A small number of doctors (endocrinologists 16.4% (27/165) vs ophthalmologists 19.4% (24/124)) preferred a treatment regimen of radioactive iodine combined with oral glucocorticoids.

**Figure 1:**
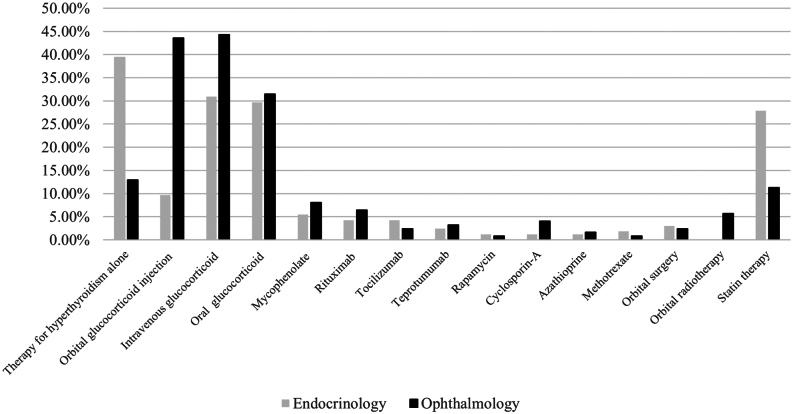
Treatment recommendations for the mild active TED patients with hyperthyroidism by departments. TED, thyroid eye disease.

When it comes to managing mild TED, the majority of endocrinologists believed that the most suitable treatment was to control hyperthyroidism and monitor continuously, while ophthalmologists reported a lower proportion (endocrinologists 39.4% (65/165) vs ophthalmologists 12.9% (16/124), *P <* 0.001). However, a larger percentage of ophthalmologists recommend more aggressive therapy compared to endocrinologists, including intravenous corticosteroid therapy (ophthalmologists 43.6% (54/124) vs endocrinologists 30.9% (51/165), *P <* 0.05) and orbital glucocorticoid injection (ophthalmologists 44.4% (55/124) vs endocrinologists 9.7% (16/165), *P <* 0.001). The use of oral corticosteroid (endocrinologists 29.7% (49/165) vs ophthalmologists 31.5% (39/124)) was relatively consistent between the two specialties. Statin therapy was not frequently recommended, but endocrinologists had a higher proportion recommending it (endocrinologists 27.9% (46/165) vs ophthalmologists 11.3% (14/124), *P <* 0.001) (Fig. 1).

##### Moderate to severe active TED

**Figure 2:**
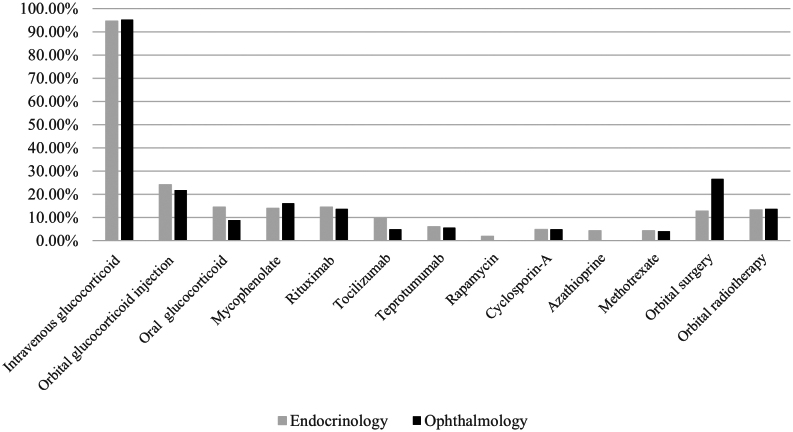
First-line treatment recommendations for the moderate-to-severe active TED patients by departments. TED, thyroid eye disease.

When inquired about the preferred first-line treatment for moderate-to-severe active TED, both endocrinologists (94.6%, 156/165) and ophthalmologists (95.2%, 118/124) overwhelmingly favored high-dose intravenous corticosteroid therapy (Fig. 2). Before and after initiating intravenous corticosteroid therapy, doctors should evaluate various indicators to prevent adverse reactions. The most commonly assessed indicators included complete blood count (91.7%, 265/289), liver function (93.1%, 269/289), kidney function (87.5%, 253/289), and blood glucose level (87.5%, 253/289). Additionally, a substantial number of endocrinologists believed that routine checks of bone density (75.2%, 124/165) and blood pressure (75.8%, 125/165) were also necessary.

The most commonly employed protocol was 0.5 g/week intravenous methylprednisolone for 6 consecutive weeks followed by 0.25 g/week for 6 weeks (endocrinologists 52.7% (87/165) vs ophthalmologists 55.7% (69/124)). Moreover, a minority of doctors preferred to complement the above conventional protocol with oral mycophenolate (endocrinologists 13.3% (22/165) vs ophthalmologists 14.5% (18/124)), suggesting that the treatment had not been widely recognized. Additionally, some doctors opted for a treatment regimen of 0.5–1 g/day intravenous methylprednisolone daily or every other day (repeat three times for 1–2 weeks), followed by tapering dose of oral prednisolone (endocrinologists 22.4% (37/165) vs ophthalmologists 25.8% (32/124)), which reported a lesser proportion than the 12-week protocol.

If there was a poor response to intravenous corticosteroid therapy, both endocrinologists and ophthalmologists commonly recommended a second-line treatment of orbital radiotherapy in combination with immunosuppressants. Moreover, ophthalmologists (51.6%, 64/124) had a higher proportion of recommending this approach compared to endocrinologists (37.6%, 62/165, *P <* 0.05). Ophthalmologists (25.8%, 32/124) and endocrinologists (23.0%, 38/165) had a similar ratio of recommending surgical intervention. Additionally, endocrinologists had a higher proportion of choosing repeated intravenous corticosteroids (endocrinologists 31.5% (52/165) vs ophthalmologists 15.3% (19/124), *P <* 0.01), while both specialties held a consistent perspective of selecting rituximab (endocrinologists 26.7% (44/165) vs ophthalmologists 25% (31/124)).

When it comes to treating TED patients with dysthyroid optic neuropathy (DON), both endocrinologists and ophthalmologists commonly choose 0.5–1 g/day intravenous methylprednisolone daily or every other day (repeat three times for 1–2 weeks). If there is no improvement in vision, immediate orbital surgery is conducted. Notably, the proportion of ophthalmologists selecting this treatment plan is higher (ophthalmologists 61.3% (76/124) vs endocrinologists 27.9% (46/165), *P <* 0.001). Endocrinologists reported a more option of the same dosage of intravenous methylprednisolone followed by a gradual tapering off of oral glucocorticoids (endocrinologists 23.6% (39/165) vs ophthalmologists 12.9% (16/124), *P <* 0.05). Additionally, 0.75 g/week intravenous methylprednisolone for six consecutive weeks followed by 0.5 g/week for 6 weeks was also preferred by part of endocrinologists (endocrinologists 23.0% (38/165) vs ophthalmologists 8.9% (11/124), *P <* 0.01). Both endocrinologists and ophthalmologists had a subset of doctors who advocated for immediate orbital surgery, and the proportions are relatively consistent (endocrinologists 19.4% (32/165) vs ophthalmologists 14.5% (18/124)).

##### TED in special situation

If moderate-to-severe inactive TED patients perceived that TED had a significant impact on their daily life and sought treatment, ophthalmologists (70.2%, 87/124) had a significantly higher preference for orbital surgery compared to endocrinologists (28.5%, 47/165, *P <* 0.001). The treatment preference of orbital surgery meets the patient’s desire for improved quality of life. Conversely, endocrinologists (43.6%,72/165) had a higher inclination toward selecting intravenous corticosteroid therapy compared to ophthalmologists (17.7%, 22/124, *P <* 0.001), suggesting there was a misconception about the corticosteroid use.

When TED occurs in conjunction with systemic diseases such as diabetes and hypertension, concurrent treatment becomes necessary. In cases where moderate-to-severe active TED was accompanied by diabetes and poor control of blood glucose, the majority of endocrinologists (80%, 132/165) opted for a combination of antidiabetic medications and intravenous corticosteroid therapy. This preference was significantly higher compared to ophthalmologists (49.2%, 61/124, *P <* 0.001).

#### Prospects and barriers for use of biological agent

In recent years, the emergence of various biological agents has brought new hope for patients with TED. However, several issues need to be addressed before these agents can be widely implemented in clinical practices.

**Figure 3:**
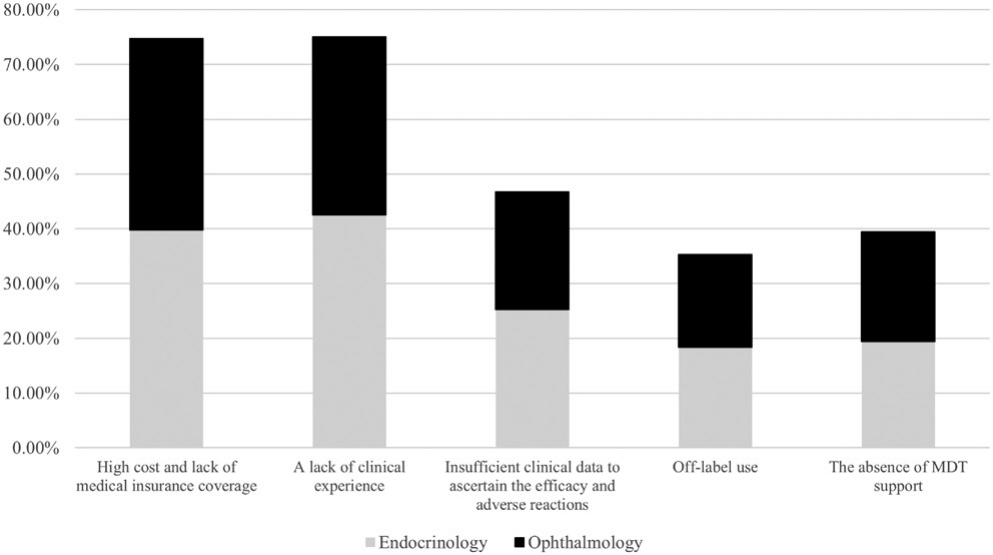
Barries for the use of the biological agents as the first-line treatment for moderate-to-severe active TED patients. MDT, multidisciplinary treatment; TED, thyroid eye disease.

According to the survey, a significant hurdle identified by 74.7% (216/289) of doctors was the high cost and lack of medical insurance coverage. Additionally, 75.1% (217/289) of doctors expressed a lack of clinical experience. Concerns were also raised regarding insufficient clinical data to ascertain efficacy and adverse reactions (46.7%, 135/289), off-label use (35.3%, 102/289), and the absence of MDT support (39.5%, 114/289) (Fig. 3).

Currently, a domestic IGF-1R inhibitor is undergoing clinical trials in China and is expected to become available in the near future. In this context, 45.3% (131/289) of participants expressed their willingness to utilize domestic IGF-1R inhibitor as a first-line treatment for TED, while 8.3% (24/289) held opposing views, and the remaining 46.4% (134/289) of doctors maintained a cautious approach, adopting a wait-and-see attitude.

#### Challenges and concerns about the management of TED

The management of TED in China still faces numerous challenges. The primary concerns among doctors included the absence of predictive biomarkers and appropriate preventive measures (78.6%, 227/289), the difficulty in fully restoring to their pre-TED level despite treatment (68.9%, 199/289), and the limited availability of imaging techniques for objective assessment of TED activity and severity (64.0%, 185/289).

#### International difference in the management

In American and European countries, 47.1% (107/227) of endocrinologists had an MDT pattern in their hospital, but the majority (85.5%, 194/227) were able to refer to ophthalmologists for TED practicing in their respective areas. The coverage of MDT (36.7%, 106/289) in China is lower compared to Western countries (*P <* 0.05), and so is the referral rate (47.4%, 137/289,* P <* 0.001).

When it comes to treating the mild active patients, the experts from the ATA and ETA strongly advocated for the referral of patients to ophthalmologists and the therapy for hyperthyroidism alone, in addition to ocular surface supportive therapies and risk factors control, which is similar to endocrinologists in China. For moderate-to-severe active patients, the most recommended treatment approach was intravenous corticosteroid in Europe and China, while in America, teprotumumab had emerged as a first-line therapeutic agent for moderate-to-severe TED patients. The main reason for the disparity is that teprotumumab is not licensed outside of America (28).

#### Assessment of concordance with 2022 Chinese TED Guidelines recommendations by departments

Table 3 summarizes the degree of concordance between survey respondents and selected recommendations from the 2022 Chinese TED Guidelines by departments. It is important to emphasize that the survey was aimed at assessing the differences in clinical practices on TED management between departments. This serves as a reminder to enhance the collaboration of MDT approach.

**Table 3:** Comparison of Chinese clinical practice guideline recommendations to survey responses.

Recommendation no.^a^	Chinese TED guideline recommendations	Survey concordance		*P*
		Endocrinologists	Ophthalmologists	
2	TED should be categorized based on its level of activity and severity.	85.36%	85.25%	NS
3	The classification of TED activity period should be based on both CAS and imaging examinations.	49.09%	62.90%	<0.05
4	It is recommended to establish a multidisciplinary approach for the diagnosis and treatment of TED	42.42%	29.03%	<0.05
5	It is suggested to assess the effectiveness of treatment for TED by combining patient self-reporting (QOL) and objective measures reported by the doctor.	72.73%	47.58%	<0.001
6	It is advised that TED patients quit smoking and control other risk factors.	63.48%^b^	33.86%^b^	<0.001
10	Patients suffering from TED should undergo continuous ocular surface support therapy throughout the entire duration of their condition.	97.58%	100%	NS
11	Intravenous glucocorticoids therapy is regarded as the primary treatment for patients with moderate, severe, and extremely severe cases of TED.	94.55%	95.16%	NS
18	For patients who have been inactive or stable for at least 6 months, surgical treatment can be chosen.	28.48%	70.16%	<0.001
21	In cases of TED complicated by DON, prompt initiation of high-dose intravenous glucocorticoid for 1–2 weeks is recommended. If there is no improvement, surgical intervention should be considered.	27.88%	61.29%	<0.001

^a^Represents the number in the Chinese guideline (13) on the diagnosis and treatment of thyroid-associated ophthalmopathy; ^b^Represents the percentage of respondents who took into account all the involved risk factors in the questionnaire at the same time.

TED, thyroid eye disease; CAS, clinical activity score; QOL, quality of life; DON, dysthyroid optic neuropathy.

## Discussion

To the best of our knowledge, this study specifically examines the divarication and agreements in the clinical management of TED among different departments and compares these findings with the recently published TED guidelines. According to the survey, there was a belief that the prevalence of TED had been on the rise in the past decade. This finding aligns with the survey on the management of TED conducted in China 8 years ago (29) but contradicts the incidence rates reported in surveys in American and European countries during the same period. ETA and ATA specialists perceived the incidence rate of TED to be relatively stable (28). However, it should be noted that there are no recent epidemiological studies confirming the trend in TED incidence in China. We postulate that this disparity could be attributed to the heightened awareness and emphasis on TED in China in recent years, potentially resulting in a greater number of diagnosed cases that may have previously gone unnoticed. In European and American countries, there has been an earlier implementation of early diagnosis and multidisciplinary management for TED, resulting in a more stable trend in the number of newly reported cases in recent years.

Moreover, based on our survey, the prevalence of smoking among patients with TED remained alarmingly high. A majority of participants (61.6%, 178/289) reported that over 25% of their newly diagnosed TED patients were smokers. Efforts should be made to enhance smoking cessation recommendations for high-risk individuals (particularly Graves’ disease patients). We endeavored to explore the widely accepted cutoff value of proptosis for the diagnosis of TED in China; however, there was a lack of consensus among healthcare professionals. The cutoff values of 18.3 mm (15.2%, 44/289) and 16 mm (24.9%, 72/289) were relatively well-recognized, albeit their approval rating remained below 25%. The definition of proptosis in the Chinese population remains a topic of ongoing controversy, and a comprehensive nationwide survey encompassing a large sample size may be necessary to ascertain an accurate cutoff value. Determining a cutoff value for the diagnosis of proptosis was an intricate task that is particularly susceptible to multiple factors, rather than being a straightforward process.

Regarding imaging techniques for TED, orbital CT and ocular MRI are the two predominant techniques employed. Endocrinologists tended to rely more heavily on orbital CT, possibly due to its wider accessibility and lower cost, whereas ophthalmologists favored ocular MRI as it offered superior visualization of soft tissue structures (30). The utilization of contrast-enhanced scans can provide additional assistance in determining the active phase of TED, which is a crucial factor in predicting the efficacy of intravenous corticosteroids and other immunosuppressants (31). In the case of patients who may require orbital surgery, standardized CT is typically the primary choice (9).

When it came to controlling hyperthyroidism in relation to TED, antithyroid drugs were the preferred treatment option. However, the proportion of antithyroid drugs was significantly lower than the data reported by Wang in 2021 in the survey on the management of hyperthyroidism (80.6% (233/289) in our survey vs 98.5% (745/756) in 2021, *P <* 0.001). Conversely, a portion of doctors (17.7% (51/289)) opted for radioiodine combined with oral glucocorticoids in our survey, which was actually higher than the 1.3% (10/756) proportion reported for radioiodine usage (*P <* 0.001) (32). This is quite perplexing, and we speculate that it may be due to the potential benefit of eradicating hyperthyroidism completely for TED, with oral glucocorticoids counteracting the exacerbating effects of radioiodine on TED (33, 34).

In the management of mild TED, Chinese doctors, particularly ophthalmologists, have opted for more aggressive treatment approaches, such as orbital corticosteroid injections. It is potentially due to patients’ desire for medical intervention to a measure. Ebner *et al*. reported that orbital injections of triamcinolone have demonstrated effectiveness in reducing diplopia and the size of soft tissues, with no significant systemic or ocular side effects (35). Similarly, Swaify *et al*. reported that triamcinolone injections have shown to decrease CAS and reduce proptosis, without any serious complications (36). Nevertheless, it is worth mentioning that the aforementioned trials focused exclusively on individuals with moderate-to-severe TED. Hence, the utilization of these high-risk treatment methods, such as orbital corticosteroid injection, in patients with mild TED should be approached with more caution.

For moderate-to-severe active TED, high-dose intravenous corticosteroid therapy remained the most recommended treatment approach among participants. Currently, the majority of doctors adhere to the standard dosage of intravenous methylprednisolone recommended in the EUGOGO guidelines. The current clinical practices have been improved compared to the clinical survey of TED conducted by Xu *et al*. in 2015 (29). This suggests a gradual standardization of the management of TED in recent years.

It is important to acknowledge that there were significant disagreements in the management of TED between the endocrinology and ophthalmology departments. Endocrinologists adhered to stricter control of risk factors, promptly employing lipid-lowering medications and selenium supplements to assist in the clinical management of TED (37, 38). Additionally, they were more inclined to treat TED after stabilizing the underlying disease (e.g. diabetes), which might be a safer approach. On the contrary, ophthalmologists were positive about surgical intervention, which might offer more benefits for inactive TED patients or those suffering from DON. They were more proficient in a series of complicated eye exams. The contradiction just exactly underscores the undeniable significance of MDT. Both endocrinologists and ophthalmologist require mutual learning and collaborative efforts for the accurate management of TED.

The joint statement published by ATA and ETA advocates for the early referral of patients to specialized MDT of TED (10, 11). The timing of referral also plays a critical role in ensuring the patients achieve satisfactory treatment outcomes (39). Unfortunately, our survey indicated that the proportion of medical institutions in China with comprehensive MDT systems was still limited, and the coverage of MDT for TED stood at 36.7%. This may be attributed to the absence of a supportive institutional framework, the complexity of patient referral processes, and inadequate communication between departments. These factors can impede the establishment of robust collaboration in the management of TED.

Despite the relatively high response rates and safety demonstrated by monoclonal antibodies in improving TED (17, 18, 19, 20, 21, 22, 23, 24), their utilization remained rare. The primary factor contributing to the condition was the low accessibility of new drugs, which were currently only available in a few large medical institutions. The deeper underlying reason behind the low accessibility rate might be the high cost of biologics. Patients from financially disadvantaged backgrounds find it difficult to afford therapies that are expensive and not covered by medical insurance. The supply of medications in healthcare institutions is affected by the needs of patients. Furthermore, the availability is not the sole reason impeding the broader use of these new drugs. Due to the short span of time of biological agent introduction, both patients and doctors harbor reservations about their efficacy and safety. There is still a certain level of uncertainty and reluctance in fully embracing and utilizing these new drugs. Going forward, it is crucial to integrate newly developed agents into clinical practice, address the doubts of the public, and ensure more individuals benefit from these new treatment options.

Our study has also identified several issues in the current management of TED in China. First, due to the unknown cutoff value of proptosis in the Chinese population, diagnostic criteria for TED have not been established. Secondly, there is a scarcity of new treatment resources, such as biologics, with only a few large medical institutions having access to them. Additionally, there are discrepancies between ophthalmology and endocrinology departments regarding the treatment of TED. Further collaboration and communication of MDT need to be established and strengthened. These issues warrant specific attention in future research and policy development in the field of TED management in China.

Our study has certain limitations. First, the number of collected questionnaires was relatively small, which brings out difficulty in accurately evaluating the clinical practices in the management of TED in China. Moreover, the absence of geography information about the participants restricted our ability to estimate and analyze potential variations and associations.

## Conclusion

This study assesses the current clinical practice patterns in the management of TED from various perspectives. Significant differences were observed between the endocrinology and ophthalmology departments, further reinforcing the importance of MDT. Our survey also highlights several issues in the management of TED in China, including contentious diagnostic criteria, inadequate treatment resources, and low coverage of the MDT pattern. Moreover, the survey concordance to guidelines remains incomplete, with insufficient consideration of risk factors and inappropriate corticosteroid usage, reminding of the necessity of updating knowledge. The effective management of TED requires not only unilateral efforts from doctors but also synergy between endocrinology and ophthalmology, advancements in technology and therapeutics, policy support, and ongoing exploration in scientific research.

## Supplementary Materials

Supplementary Table S1 Applicable Survey Items

## Declaration of interest

The authors declare that there is no conflict of interest that could be perceived as prejudicing the impartiality of the study reported.

## Funding

This study was funded by the National Natural Science Foundation of Chinahttp://dx.doi.org/10.13039/501100001809 (No. 82270833).

## Author contribution statement

YL and HZ contributed to the study design or concept, all authors contributed to the analysis and interpretation of the data. JC and YL drafted the article. All the authors critically reviewed and edited the article. All the authors approved the final version and were responsible for the decision to submit the article.
