# Supplementary material for: A Chinese survey of clinical practice on the management of thyroid eye disease
Source: Eur Thyroid J. 2024 May 23;13(3):e230269. doi: 10.1530/ETJ-23-0269 (PMC11227091; doi:10.1530/ETJ-23-0269)
Supplement: Supplementary Table S1 Applicable Survey Items [file supplementary_table_1.pdf]

Supplementary Table S 1 Applicable Survey Items

Question	Response Options
Demographics of Participants	
What is your specialty ?	Endocrinology, ophthalmology, other please specify
Which of the following describes how you think of yourself?	Male female
What best describes your primary practice?	Tertiary hospitals, secondary hospitals, community hospitals, private hospitals, other medical institutions
What is your professional title?	Chief physician, associate chief physician, attending physician, resident physician
How many years have you been practicing your specialty?	Less than 5 years, 5-10 years, 11-15 years, 16-20 years, more than 20 years
TED management	
How do you think the number of new cases of thyroid eye disease (TED)per annum has changed in the last 10 years in the area where you practice?	Unchanged, increased, decreased, not sure
What is the threshold value for assessing the proptosis in the diagnosis of TED in your clinical practice?	14mm, 16mm,18.3mm,18.6mm,20mm,22m, the patient reported increased proptosis, it is still unclear
What imaging techniques do you typically use to objectively assess the activity and severity of TED?	orbital MRI scan, orbital CT scan, orbital DOTA-PET, assessment of pupils for relative afferent pupillary defect, ocular ultrasound.
What criteria do you typically use to stage the activity of TED patients?	Clinical activity score (CAS), orbital MRI, orbital CT, clinical activity staging is generally not performed, CAS in conjunction with orbital MRI
What indicators do you typically use to grade the severity of TED patients?	EUGOGO classification, assessment of patient's quality of life, evaluation of optic nerve function, generally does not involve specific severity grading.
Does your hospital or institution have a multidisciplinary TED clinic (i.e. a clinic where patients are cared for by a coordinated team [treatment decisions as a group] that includes endocrinologists and ophthalmologists)?	Yes, no
Do you refer any of your patients to a multidisciplinary TED Clinic?	Often (>50% of my patients), sometimes (＜50% of my patients), never
When do you typically involve an ophthalmologist in the care of a patient with TED? (you may check more than one box if you wish)	Referral is done immediately upon initial diagnosis, I manage these patients myself without involving an ophthalmologist unless eye surgery is required, If TED is severe or extremely severe, If TED is active, there is no need for further referrals, this question is not applicable to me I am ophthalmologist
What is the estimated prevalence of smokers (current smokers at the time of diagnosis) in patients with TED where you practice?	0-25 %, 26-50 %, 51-75%, above 75%, I do not know
Would you recommend smoking cessation or avoiding passive smoking for patients with TED?	Yes, no
Would you recommend lipid level testing for patients with TED?	Yes, no
Would you recommend the use of lipid-lowering medications for patients with TED who have hypercholesterolemia?	Yes, no
Would you recommend appropriate vitamin D supplementation for patients with TED according to the level of vitamin D?	Yes, no
Would you recommend appropriate selenium	Yes, all TED patients receive selenium

supplementation for patients with TED?	supplementation, yes, selenium supplementation is only given to patients with mild active TED, no
Would you recommend the use of artificial tears, eye ointments, or other ocular surface support therapies for patients with TED?	Yes, no
Would you recommend patients with TED to wear glasses for vision correction or to reduce glare?	Yes, no
Would you incorporate the results of the Graves' Ophthalmopathy Quality of Life (GO-QoL) questionnaire into the assessment of treatment efficacy for TED patients?	Yes, no
There is list of treatments for patients with TED. Regardless of whether you have used these therapies, please mark which treatment options are available for your patients (at your institution)	Intravenous corticosteroids, orbital corticosteroids injection, oral corticosteroids, mycophenolate, rituximab, tocilizumab, teprotumumab, rapamycin, cyclosporin-A, azathioprine, methotrexate, orbital surgery, orbital radiotherapy
The Graves' disease patients with mild active TED and a CAS of 3 points present a management challenge. Do you have a preference regarding the first-line treatment for her hyperthyroidism?	Anti-thyroid drugs, radioiodine, radioiodine plus oral steroid prophylaxis, thyroidectomy
For the Graves' disease patients with mild active TED, what would be your treatment recommendation(s) at this stage for her TED? (you may check more than one box if you wish)	Treating hyperthyroidism alone, Intravenous corticosteroids, orbital corticosteroids injection, oral corticosteroids, mycophenolate, rituximab, tocilizumab, teprotumumab, rapamycin, cyclosporin-A, azathioprine, methotrexate, orbital surgery, orbital radiotherapy, statin therapy
For patients with moderate to severe active TED, which treatment is the first-line treatment?	Intravenous corticosteroids, orbital corticosteroids injection, oral corticosteroids, mycophenolate, rituximab, tocilizumab, teprotumumab, rapamycin, cyclosporin-A, azathioprine, methotrexate, orbital surgery, orbital radiotherapy
Before and after administering intravenous corticosteroids therapy, which indicators would you evaluate in order to prevent adverse reactions in patients? (you may check more than one box if you wish)	Complete blood count, urinalysis, liver function, kidney function, blood glucose, electrolytes, blood coagulation function, indicators related to viral and autoimmune hepatitis, blood pressure, electrocardiogram, chest X-ray or CT scan, bone density measurement
What is your most commonly used corticosteroids dosage for moderate to severe active TED patients receiving intravenous corticosteroids treatment?	0.5 g/week intravenous methylprednisolone for six consecutive weeks followed by 0.25 g/week for six weeks, 0.5 g/week intravenous methylprednisolone for six consecutive weeks followed by 0.25 g/week for six weeks plus oral mycophenolate, 0.75 g/week intravenous methylprednisolone for six consecutive weeks followed by 0.5 g/week for six weeks, 0.5-1 g/d intravenous methylprednisolone for three consecutive days (repeat 3 times for 1-2weeks), followed by tapering dose of oral prednisolone
What is your preferred treatment approach for TED patients with dysthyroid optic neuropathy (DON)?	0.75 g/week intravenous methylprednisolone for six consecutive weeks followed by 0.5 g/week for six weeks, 0.5-1 g/d intravenous methylprednisolone for three consecutive days (repeat 3 times for 1-2weeks), followed by tapering dose of oral prednisolone, urgent eye surgery, elective eye surgery, 0.5-1 g/d intravenous methylprednisolone for three consecutive days (repeat 3 times for 1-2weeks), conduct eye surgery if no improvement, Tocilizumab as the initial treatment, conduct eye surgery if no improvement
Patient has Type 2 Diabetes with poor control of blood glucose levels. What treatment(s) would you institute as first line for her TED? (you may check more than	Antidiabetic medication plus intravenous corticosteroids, orbital corticosteroids injection, oral corticosteroids, mycophenolate, rituximab,

one if you wish)	tocilizumab, teprotumumab, rapamycin, cyclosporin-A, azathioprine, methotrexate, orbital surgery, orbital radiotherapy
The patient with non-active moderate-to-severe subjectively perceive a significant impact on their quality of life. What treatment(s) would you institute or recommend? (you may check more than one if you wish)	Intravenous corticosteroids, orbital corticosteroids injection, oral corticosteroids, mycophenolate, rituximab, tocilizumab, teprotumumab, rapamycin, cyclosporin-A, azathioprine, methotrexate, orbital surgery, orbital radiotherapy
The patient with active moderate-to-severe obtains no obvious improvement after intravenous corticosteroids. What second-line treatment(s) would you institute or recommend? (you may check more than one if you wish)	Repeated intravenous corticosteroids, orbital corticosteroids injection, oral corticosteroids plus orbital radiotherapy, orbital radiotherapy, orbital radiotherapy plus immunosuppressor, mycophenolate, rituximab, tocilizumab, teprotumumab, rapamycin, cyclosporin-A, azathioprine, methotrexate, orbital surgery
When considering biological agents (rituximab, tocilizumab, teprotumumab) as a first line therapy for patients with moderate and active TED, what do you think are barriers for its use? (you may check more than one if you wish)	high cost and lack of medical insurance coverage, a lack of clinical experience, insufficient clinical data to ascertain the efficacy and adverse reactions, off-label use, the absence of MDT support
If Teprotumumab (or domestic similar drugs) would be approved and available in China, would you use Teprotumumab as a first choice?	Yes, no, not sure
How frequently do you typically follow-up patients with moderate to severe active TED during treatment? (you may check more than one if you wish)	Every week, every month, every 3 months, at the midpoint of the treatment course, at the conclusion of the treatment course
What do you think are the difficulties physicians may face during the care of TED patients currently? (you may check more than one if you wish)	It is difficult to predict if TED develops, absence of suitable preventive measures for TED, lack of widely available imaging techniques for objective evaluation of TED activity and severity, the appearance of the eyes rarely reverts to the pre-TED level in spite of treatment, absence of recent clinical guidelines for treatment of TED, costly treatments for TED are often not covered by insurance, lack of TED multidisciplinary treatment center in my institution
